# Diagnosis of primary lung cancer and benign pulmonary nodules: a comparison of the breath test and 18F-FDG PET-CT

**DOI:** 10.3389/fonc.2023.1204435

**Published:** 2023-06-02

**Authors:** Xiangxiang Ding, Guihu Lin, Peiyu Wang, Haibin Chen, Nan Li, Zhi Yang, Mantang Qiu

**Affiliations:** ^1^ Key Laboratory of Carcinogenesis and Translational Research (Ministry of Education/Beijing), Department of Nuclear Medicine, Peking University Cancer Hospital & Institute, Beijing, China; ^2^ Key Laboratory for Research and Evaluation of Radiopharmaceuticals (National Medical Products Administration), Department of Nuclear Medicine, Peking University Cancer Hospital & Institute, Beijing, China; ^3^ Department of Thoracic Surgery, Aerospace 731 Hospital, Beijing, China; ^4^ Department of Thoracic Surgery, Peking University People’s Hospital, Beijing, China; ^5^ Thoracic Oncology Institute, Peking University People’s Hospital, Beijing, China; ^6^ Breax Laboratory, PCAB Research Center of Breath and Metabolism, Beijing, China

**Keywords:** lung cancer, breath test, pulmonary nodule, PET-CT, early detection

## Abstract

With the application of low-dose computed tomography in lung cancer screening, pulmonary nodules have become increasingly detected. Accurate discrimination between primary lung cancer and benign nodules poses a significant clinical challenge. This study aimed to investigate the viability of exhaled breath as a diagnostic tool for pulmonary nodules and compare the breath test with 18F-fluorodeoxyglucose (18F-FDG) positron emission tomography (PET)–computed tomography (CT). Exhaled breath was collected by Tedlar bags and analyzed by high-pressure photon ionization time-of-flight mass spectrometry (HPPI-TOFMS). A retrospective cohort (n = 100) and a prospective cohort (n = 63) of patients with pulmonary nodules were established. In the validation cohort, the breath test achieved an area under the receiver operating characteristic curve (AUC) of 0.872 (95% CI 0.760–0.983) and a combination of 16 volatile organic compounds achieved an AUC of 0.744 (95% CI 0.7586–0.901). For PET-CT, the SUVmax alone had an AUC of 0.608 (95% CI 0.433–0.784) while after combining with CT image features, 18F-FDG PET-CT had an AUC of 0.821 (95% CI 0.662–0.979). Overall, the study demonstrated the efficacy of a breath test utilizing HPPI-TOFMS for discriminating lung cancer from benign pulmonary nodules. Furthermore, the accuracy achieved by the exhaled breath test was comparable with 18F-FDG PET-CT.

## Introduction

The National Lung Screening Trial (NLST) has confirmed that low-dose computed tomography (LDCT) lung cancer screening can decrease lung cancer mortality by 20% in high-risk populations compared to X-ray (1). Since then, LDCT has been recommended for lung cancer screening and many pulmonary nodules have been detected along with lung cancer screening (2–4). Pulmonary nodules are defined as pulmonary lesions less than 3 cm in CT images. The pathology of pulmonary nodules includes malignant diseases, such as primary lung cancer, distant metastases, or rarer lymphoma, as well as benign causes, such as tuberculosis, pneumonia, fungi infections, and primary benign tumors (hamartoma, angioma, etc.) (5). Discriminating between primary lung cancer and benign nodules is a clinical challenge for radiologists, thoracic surgeons, and physicians practicing with LDCT-based lung cancer screening (6, 7).

Several clinical associations have made guidelines or recommendations to manage pulmonary nodules, such as the American College of Chest Physicians and The Fleischner Society in 2013 and 2017, respectively (3, 8). Rather than biopsy or surgery, high-risk pulmonary nodules are often recommended for positron emission tomography (PET) first, since it is non-invasive and can provide information for differential diagnosis and staging at the same time (9, 10). However, PET-CT is very expensive and not sensitive enough for small pulmonary nodules.

Human breath includes thousands of volatile organic compounds (VOCs) (11, 12), and mounting evidence has proved that testing VOCs of exhaled breath can precisely detect lung cancer (13–15). Cancer cells have altered metabolism and generate a variety of aberrant metabolites, and some of these aberrant metabolites could be exhaled outside and detected by mass spectrometry or nano-sensors. Breath test is totally non-invasive and easy to collect, which is a promising tool for lung cancer early detection and screening. It has been reported that VOCs in exhaled breath can discriminate lung cancer from benign nodules (16). Gas chromatography–mass spectrometer (GC-MS) has been considered as the gold standard of exhaled breath VOC analysis, but it is not applicable in clinical practice because it requires complex sample pretreatment and time- consuming detecting processes (17). Several researchers have tried to diagnose pulmonary nodules by breath test, and they used different methods (18) (19, 20). High-pressure photon ionization time-of-flight mass spectrometry (HPPI-TOFMS) is a direct mass spectrometry that does not require sample pretreatment and only takes 1 min to analyze one sample; thus, HPPI-TOFMS is a suitable tool for clinical application (13, 17). HPPI-TOFMS has been reported to be effective for detection of lung cancer (13), esophageal cancer (21), and tuberculosis (22), but it is unknown whether HPPI-TOFMS could discriminate lung cancer from benign pulmonary nodules.

In this study, we first performed the breath test by HPPI-TOFMS and trained a model in a retrospective cohort and then tested whether this model could discriminate lung cancer from benign pulmonary nodules and compared its diagnostic accuracy with PET-CT in a prospective cohort.

## Materials and methods

### Participant’s recruitment and study design

This study was reported according to the Standards for Reporting Diagnostic Accuracy, and a checklist was attached (23). The prospective specimen collection, retrospective blinded evaluation design (24) was utilized. This study was approved by the Ethics Committee Board of Peking University People’s Hospital (2021PHB349), and informed consent was obtained from all participants.

For the retrospective cohort, patients who received thoracic surgery or endobronchial ultrasound–guided transbronchial needle aspirate were consecutively recruited at the Department of Thoracic Surgery, Peking University People’s Hospital from September 2021 to October 2021. The inclusion criteria were 1) age > 18 years, 2) pulmonary lesions <3 cm in CT images, 3) the pathological diagnoses were primary lung cancer or benign lung diseases, and 4) no history of cancer and no anticancer treatment before.

For the prospective cohort, patients with pulmonary lesions planning to receive 18F-FDG PET-CT were prospectively recruited at the Department of Nuclear Medicine, Cancer Hospital of Peking University from November 2021 to January 2022. Patients were consecutively recruited with the following criteria: 1) age > 18 years, 2) with pulmonary lesions and plan to have 18F-FDG PET-CT scanning, and 3) no history of cancer within 5 years. After PET-CT scanning, we followed up pathologic diagnoses of all eligible participants. Patients who met the following criteria were excluded: 1) the lung lesions were metastasized from other organs; 2) lung lesions were larger than 3 cm in CT scans; and 3) no pathological diagnosis. For all participants, the clinical data and demographic data were collected from medical records and questionnaires.

### Exhaled breath collection

Exhaled breath samples were collected by trained investigators following the same protocol according to our previous studies (13). Exhaled breath was collected before PET-CT scanning and the morning before surgery. All participants fasted for at least 6 h before sample collection. To reduce potential confounding factors, all participants were asked not to ingest spicy food, alcohol, or coffee the night before exhaled breath collection. Disposable face masks and Tedlar bags were used to collect exhaled breath. A disposable face mask was replaced before each collection to avoid cross- contamination. Briefly, participants first gargled with pure water and then performed a single deep nasal inhalation followed by complete exhalation via their mouth into a Tedlar air bag. At both clinical centers, breath samples were collected in a fixed room and the room air was also collected before and after sample collection of participants. All air bags were delivered to lab and detected within 4 h.

### High-pressure photon ionization time-of-flight mass spectrometry detection

Exhaled breath was detected by HPPI-TOFMS as previously described (13). The pressure in the HPPI ion source was set at 500 Pa, and two capillaries were arranged in the ion source. The gas-phase exhaled breath sample was directly introduced into the ionization region through a 250- μm i.d. 0.60- m- long stainless- steel capillary from the Tedlar bag. In order to eliminate condensation of exhaled VOCs and minimize possible surface adsorption, the stainless steel capillary was heated to 100°C and the HPPI ion source was heated to 60°C. The TOF signals were recorded by a 400ps time-to-digital converter rate at 25 kHz, and all the mass spectra were accumulated for 60s. Mass spectrum peaks detected by HPPI-TOFMS with m/z <500 were recorded and 32,500 features were extracted from the HPPI-TOFMS data of each exhaled breath sample.

### Positron emission tomography–computed tomography imaging

18F-FDG PET-CT was performed as previously reported (25). The patients were instructed to fast for 6 h before 18F-FDG injection. The 18F was manufactured by HM-20 medical cyclotron (Sumitomo Corporation, Japan), and the 18F-FDG was administrated intravenously according to the patient’s body weight (3.0–3.7 MBq/kg). Imaging was performed using a PET/CT scanner (Biograph64, SIEMENS, Erlangen, Germany) operated in 3D Flow Motion (bed entry speed 1 mm/s) from the apex of the skull to the midthigh, with a PET axial field of view of 21.6 cm. The PET images were reconstructed by the TrueX + TOF method offered by the vendor. The injected activity was 3.7 MBq/kg, and the time from injection to scan was approximately 60 ± 10 min. Diagnoses of 18F-FDG PET-CT were made by two authors independently based on SUVmax and CT images. Discernments between two authors were solved by discussion.

### Statistical analyses

Machine learning models with mass spectrometry data of exhaled breath as input were constructed with the caret package (https://cran.r-project.org/web/packages/caret/). Then, the diagnostic capacity of the breath model and 16 VOCs model was evaluated with the area under the receiver operating characteristic (ROC) curve (AUC) and accuracy through the R packages pROC (https://cran.r-project.org/web/packages/pROC/index.html) in discriminating lung cancer from benign nodules.

Sensitivity, specificity, accuracy, the positive predict value, and the negative predictive value were calculated to evaluate the diagnostic performance of PET-CT and the breath test. The ROC was performed and the AUC was calculated to evaluate the diagnostic performance of PET-CT and the breath test. Baseline characteristics were analyzed with the independent t test or Fisher’s exact test. Two-sided P values less than 0.05 were considered statistically significant. All statistical analyses were performed using SPSS software (version 24.0).

## Results

### Clinical characteristics of enrolled patients

**Figure 1:**
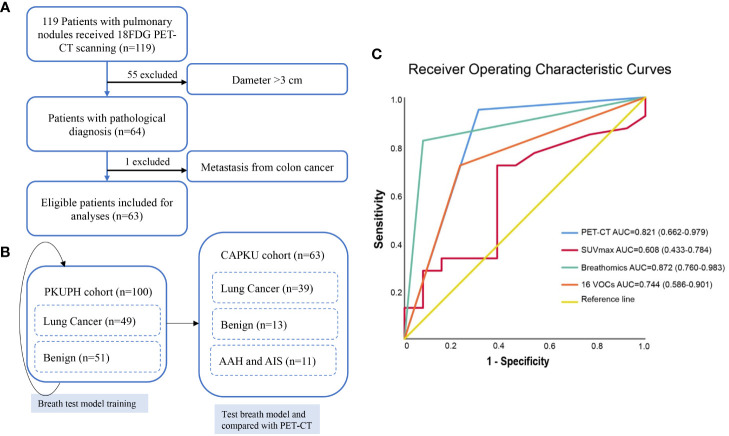
Flow diagram of participant recruitment in the Cancer Hospital of Peking University (CAPKU) cohort **(A)** and data analyses process **(B)**. Receiver operating characteristics of the breath test and positron emission tomography–computed tomography in the validation CAPKU cohort **(C)**. AAH, atypical adenomatous hyperplasia; AIS, adenocarcinoma in situ.

The overall study design is shown in **Figure 1**. We first retrospectively selected 49 lung cancer patients and 51 benign pulmonary nodules from Peking University People’s Hospital as the discovery cohort (PKUPH cohort). Then, 119 patients with pulmonary nodules and received 18F-FDG PET-CT were prospectively recruited from the Cancer Hospital of Peking University. Following this, 119 patients with pulmonary nodules who underwent 18F-FDG PET-CT were prospectively recruited from the Cancer Hospital of Peking University (CAPKU cohort) (**Figure 1A**, CAPKU cohort). Out of the 119 patients, 56 were excluded based on the exclusion criteria. A total of 63 patients were included as the validation set (**Figure 1A**, CAPKU enrollment diagram).

Detailed clinical characteristics of eligible participants are shown in **Table 1**. As shown, lung cancer patients in the PKUPH cohort were all at stage I, and in the CAPKU cohort 76.9% (30/39, **Figure 1B**), lung cancer patients were at stage I.

**Table 1:** Baseline characteristics of included participants.

	PKUPH Cohort			CHPKU Cohort		
	Lung cancer	Non-cancer lesions	*P*	Lung cancer	Non-cancer lesions*	*P*
Number of participants	49	51		39	24	
Gender			0.07			0.16
Male n (%)	20 (38.3%)	24 (48.3%)		20 (47.4%)	9 (67.9%)	
Female n (%)	29 (61.7%)	27 (51.7%)		19 (52.6%)	15 (32.1%)	
Age	54.8 ± 11.3	55.4 ± 11.3	<0.001	56.0 ± 9.8	55.9 ± 10.4	0.67
Smoking			0.963			0.188
Ever-smokers n (%)	8 (21.7%)	9.00%		16 (21.1%)	7 (39.3%)	
Never-smokers n (%)	41 (78.3%)	42 (78.5%)		23 (78.9%)	17 (60.7%)	
Pathology n (%)						
AD	49 (100%)			19 (100%)		
SCC				2		
SCLC				2		
TNM stage n (%)						
I	100 (100%)			30 (89.5%)		
II				2		
III				7 (5.3%)		

*Atypical adenomatous hyperplasia and adenocarcinoma in situ were classified as benign in this table. AD, adenocarcinoma; SCC, squamous cell carcinoma; SCLC, small cell lung cancer.

### Accuracy of high-pressure photon ionization time-of-flight mass spectrometry

HPPI-TOFMS has been proved to be accurate for lung cancer detection in a proof-of-concept study. In this study, we first trained a model to discriminate lung cancer from benign pulmonary nodules in the PKUPH cohort. A total of 100 patients with pulmonary nodules were included. In the discovery cohort, the random forest model reached the best AUC and accuracy. By constructing a model with all features of mass spectrometry peaks, the random forest model reached 82.1% sensitivity, 92.3% specificity, 84.6% accuracy, 97.0% positive predictive value, 63.2% negative predictive value, and 0.872 AUC (95% CI 0.760–0.983) in the CAPKU cohort (**Figure 1C**).

With perioperative sampling, 16 VOCs have been identified as potential lung cancer–specific biomarkers (26). Using the 16 VOCs, the model reached 71.8% sensitivity, 76.9% specificity, 73.1% accuracy, 90.3% positive predictive value, 47.6% negative predictive value, and 0.744 AUC (95% CI 0.586–0.901) in the validation cohort (**Figure 1C**).

### Accuracy of 18F-FDG positron emission tomography–computed tomography

We first analyzed the diagnostic accuracy of SUVmax in the CAPKU cohort. As shown in **Figure 1C**, SUVmax reached the best AUC of 0.608 (95% CI 0.433–0.784) when the threshold was set as 1.35. SUVmax achieved 71.8% sensitivity, 61.5% specificity, 69.2% accuracy, 84.8% positive predictive value, and 42.1% negative predictive value in the validation cohort.

When combined with CT features, 18F-FDG PET-CT had a better AUC (0.821, 95% CI 0.662–0.979) for discriminating lung cancer from benign nodules (**Figure 1C**). 18F-FDG PET-CT achieved 94.9% sensitivity, 69.2% specificity, 88.5% accuracy, 90.2% positive predictive value, 81.5% negative predictive value, and 0.744 AUC (95% CI 0.586-0.901) in the validation cohort.

### Breath test and 18F-FDG positron emission tomography–computed tomography for detection of atypical adenomatous hyperplasia/adenocarcinoma *in situ*

**Figure 2:**
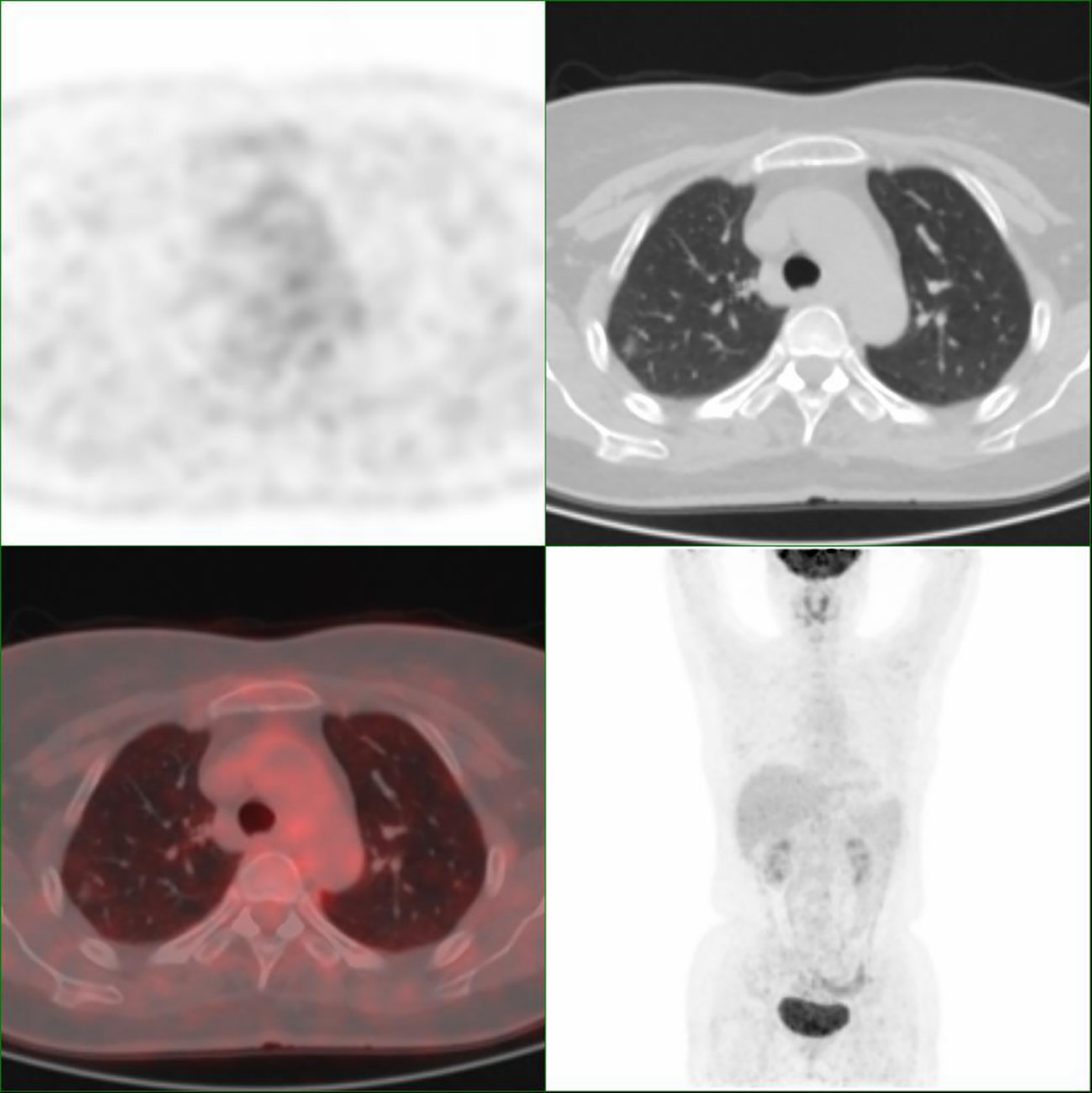
Representative images of Patient 4 with adenocarcinoma *in situ*. The nodule has an SUVmax of 0.9.

According to the current WHO classification, AAH and AIS have been classified as benign. However, AAH and AIS are the very early stage of lung adenocarcinoma; they may slowly progress and become invasive adenocarcinoma. Thus, in the validation cohort, we compared diagnostic accuracy of breath test and 18F-FDG PET-CT among pulmonary nodules diagnosed with AAH and AIS. As shown in **Table 2**, the mean SUVmax of the 11 nodules were 0.95 ranging from 0.4 to 3.2, with only one patient having nodules with SUVmax > 1.35. A representative image was shown in **Figure 2**. Among 11 patients with AAH/AIS, 4 patients were correctly detected by the breath test and 7 patients were classified as lung cancer according to SUVmax.

**Table 2:** Detailed diagnoses by breath test and PET-CT among 11 participants with atypical adenomatous hyperplasia or adenocarcinoma *in situ*.

ID	Pathology	Prediction based on all Breathomics data	Prediction based on 16 VOCs	Prediction based on PET-CT	SUVmax
Patient 1	AAH	Benign	Benign	Benign	0.7
Patient 2	AIS	Benign	Lung cancer	Lung cancer	1
Patient 3	AIS	Benign	Benign	Benign	0.9
Patient 4	AIS	Benign	Lung cancer	Lung cancer	0.9
Patient 5	AIS	Benign	Lung cancer	Lung cancer	0.8
Patient 6	AIS	Lung cancer	Benign	Lung cancer	0.9
Patient 7	AIS	Benign	Benign	Benign	0.5
Patient 8	AIS	Benign	Benign	Lung cancer	0.5
Patient 9	AIS	Lung cancer	Lung cancer	Benign	0.4
Patient 10	AIS	Benign	Benign	Lung cancer	3.2
Patient 11	AIS	Benign	Benign	Lung cancer	0.7

## Discussion

In this study, we found that SUVmax had the best diagnostic performance when the cutoff value was set as 1.35, which is much lower than the previously reported 2.5 (27, 28). It has been reported that 18F-FDG PET-CT could detect small pulmonary nodules less than 1 cm, lower cutoff may provide accurate diagnosis of benign and malignant pulmonary nodules (29). However, it should be noted that lung cancer risk among patients with PET-negative pulmonary nodules cannot be neglected. Akpoviroro et al. followed up 191 LungRADS-4 patients, and they found that 22.4% (15/67) patients were diagnosed with lung cancer in the PET-negative group (30).

In this study, SUVmax alone achieved an AUC of 0.608; however, the AUC increased to 0.821 when CT image features were added, indicating an improvement in diagnostic accuracy. Among 11 patients with AAH or AIS, 7 were correctly diagnosed as lung cancer by 18F-FDG PET-CT. As the mean SUVmax was 0.95 and only one nodule had SUVmax > 1.35, SUVmax alone is not sufficient for the detection of AIS/AAH. Furthermore, the inclusion of CT images significantly enhanced the accuracy of diagnosis in 18F-FDG PET-CT. These data suggested that SUVmax alone is not enough to discriminate lung cancer from benign pulmonary nodules and CT images should be an indispensable part of pulmonary nodule follow-up.

Compared with PET-CT, the breath test showed better diagnostic accuracy in the CAPKU cohort. The 16 VOCs achieved 71.8% sensitivity and 76.9% specificity, while all exhale breath mass spectrometry features achieved 82.1% sensitivity and 92.3% specificity. Although the diagnostic accuracy decreased slightly, the 16 VOCs still achieved accuracy >70%. These data suggest that the breath test may be used for diagnosis of pulmonary nodules or follow-up, especially for PET-negative pulmonary nodules.

Discriminating lung cancer from benign pulmonary nodules is very challenging, and many methods have been tried, such as circulating cell-free DNA, metabolomics, and exhaled breath. Thus, this study provides new insights into the current lung cancer screening strategy. Exhaled breath is easy and non-invasive to collect, and the breath test by HPPI-TOFMS is fast and feasible, which is very useful to help identify high-risk populations. A well-designed study is warned to investigate how to integrate the breath test into the current LDCT lung cancer screening model.

It is crucial to note the limitations of the study when interpreting the results. While over 90% of incidentally detected pulmonary nodules are generally benign, participants in this study were highly selected before they were recruited, which resulted in a lower proportion of benign nodules. On the other hand, although we have identified 16 lung cancer– specific VOCs in a previous study (26), we did not compare results from HPPI-TOFMS with the current gold- standard GC-MS as reported by Markar et al. (31).

## Conclusions

In summary, the study demonstrated the efficacy of a breath test utilizing HPPI-TOFMS for discriminating lung cancer from benign pulmonary nodules, and the accuracy achieved by the exhaled breath test was comparable with 18F-FDG PET-CT.

## Data availability statement

The original contributions presented in the study are included in the article/supplementary material. Further inquiries can be directed to the corresponding authors.

## Ethics statement

The studies involving human participants were reviewed and approved by Ethics Committee Board of Peking University People’s Hospital. The patients/participants provided their written informed consent to participate in this study.

## Author contributions

Conceptualization, XD, NL, ZY, and MQ; methodology, XD, PW, and GL; software, HC; formal analysis, XD; investigation, XD, HC, NL, and MQ; writing—original draft preparation, XD and MQ; writing—review and editing, XD, NL, ZY, and MQ; visualization, XD and MQ; supervision, NL, ZY, and MQ; project administration, NL, ZY, and MQ; funding acquisition, MQ. All authors have read and agreed to the published version of the manuscript.
